# Reduced circulating 20-hydroxyeicosatetraenoic acid and ACE2 in acute coronary syndrome with exploratory cell-based observations

**DOI:** 10.1038/s41598-026-56316-9

**Published:** 2026-06-10

**Authors:** Ieva Čiapienė, Aistė Ivanauskienė, Nora Kupstytė-Krištaponė, Vaiva Lesauskaitė, Robertas Pranevičius, Gintarė Šakalytė, Daiva Stanislovaitienė, Cynthia Sithole, Vacis Tatarūnas

**Affiliations:** 1 Institute of Cardiology, Lithuanian University of Health Sciences, Sukileliu 15, Kaunas, LT, 50162 Lithuania; 2 Heart Center, Medical Academy, Lithuanian University of Health Sciences, Eiveniu 2, Kaunas, LT, 50161 Lithuania; 3 Department of Ophthalmology, Lithuanian University of Health Sciences, Eiveniu 2, Kaunas, LT, 50161 Lithuania; 4 Department of Physiology, School of Medicine, Faculty of Health Sciences, University of Pretoria, Private Bag X323, Gezina, 0031 Pretoria South Africa

**Keywords:** 20-HETE, ACE2, ischemic heart disease, renin-angiotensin system, stable coronary artery disease, acute coronary syndrome, Biomarkers, Cardiology, Diseases, Medical research

## Abstract

Progression of ischemic heart disease to acute coronary syndrome (ACS) is fueled by endothelial dysfunction and disruptions in vascular signaling pathways. The relationship between the angiotensin-converting enzyme 2 (ACE2)/renin-angiotensin system (RAS) axis and eicosanoid metabolism is still not fully understood. This study aimed to investigate whether changes in circulating ACE2 and 20-hydroxyeicosatetraenoic acid (20-HETE) reflect dysregulation of a combined ACE2/RAS–eicosanoid axis in ACS and to evaluate the diagnostic and functional significance of 20-HETE. We studied 122 patients with ischemic heart disease (including stable coronary artery disease (SCAD) and ACS) and 54 controls. Levels of circulating ACE2 and 20-HETE were measured using ELISA. Additionally, in vitro experiments with cell culture models were conducted to evaluate how pharmacological modulation of the ACE2/RAS axis affects nitric oxide (NO) production, inflammatory signaling, and CYP4F2 expression. Both ACE2 and 20-HETE levels were significantly lower in ACS compared to SCAD and controls (*p* < 0.001). Circulating 20-HETE effectively distinguished ACS from SCAD with an AUC of 0.91. In endothelial cells, altering the ACE2/RAS axis impacted NO availability and inflammatory gene expression, correlating with changes in *CYP4F2* levels. 20-HETE affected cellular metabolic activity and GPR75 signaling, indicating a functional connection between RAS signaling and eicosanoid pathways. Lower circulating 20-HETE levels are associated with disruption of the integrated ACE2/RAS–eicosanoid axis, which is linked to endothelial dysfunction in ACS. These results suggest that 20-HETE may serve not only as a biomarker but also as part of a broader vascular signaling network related to acute coronary instability.

**Supplementary Information:**

The online version contains supplementary material available at 10.1038/s41598-026-56316-9.

## Introduction

Ischemic heart disease (IHD) remains the leading cause of cardiovascular mortality worldwide and continues to impose a substantial global health burden^1–3^. IHD encompasses a spectrum of conditions ranging from chronic stable coronary disease to acute coronary syndrome (ACS). ACS typically results from a sudden disruption of an atherosclerotic plaque followed by thrombotic coronary occlusion and myocardial ischemia^4^. Although significant progress has been made in cardiovascular diagnostics and treatment, the early identification of patients progressing from stable coronary artery disease (SCAD) to acute coronary events continues to present a substantial clinical challenge.

The pathophysiology of ACS is characterized by intricate interactions among endothelial dysfunction, vascular inflammation, oxidative stress, and abnormal vascular signaling pathways^5–7^. Several circulating biomarkers that reflect vascular inflammation or plaque instability, such as high-sensitivity C-reactive protein (hs-CRP), matrix metalloproteinases, and myeloperoxidase (MPO), have been investigated as potential predictors of acute coronary events^8^. Nevertheless, these biomarkers demonstrate limited diagnostic accuracy in differentiating ACS from SCAD.

Among the key regulatory systems involved in vascular homeostasis, the renin-angiotensin system (RAS) plays a central role. It modulates vascular tone, inflammatory signaling, and endothelial function^9,10^. Angiotensin-converting enzyme 2 (ACE2), a key component of the alternative RAS axis, counteracts the effects of angiotensin II by generating angiotensin-(1–7) and provides protective effects against vascular inflammation, oxidative stress, and impaired endothelial function. Dysregulation of the RAS pathway has been implicated in the development of endothelial dysfunction and atherosclerotic plaque progression, processes that are critically involved in the pathogenesis of acute coronary events^11^. In parallel to RAS signaling, arachidonic acid metabolites have emerged as important regulators for vascular homeostasis and cardiovascular function. Among these metabolites, 20-hydroxyeicosatetraenoic acid (20-HETE) plays a key role in the regulation of vascular tone and endothelial function. 20-HETE is produced by cytochrome P450 (such as CYP4A and CYP4F) enzymes in vascular smooth muscle and endothelial cells. This lipid mediator functions as a potent vasoactive molecule that induces vasoconstriction, modulates sodium transport, and regulates blood pressure. Furthermore, 20-HETE is involved in pro-inflammatory signaling, oxidative stress generation, and platelet activation, thereby establishing a connection between arachidonic acid metabolism, vascular inflammation, and thrombotic processes^12–15^.

The biological rationale for studying ACE2/RAS and 20-HETE together lies in their shared impact on endothelial function, vascular tone, oxidative stress, and inflammation. The activation of RAS can lead to increased oxidative stress through angiotensin II, which in turn reduces the availability of nitric oxide (NO). On the other hand, eicosanoids derived from CYP450, such as 20-HETE, play a role in regulating vascular reactivity and may enhance inflammatory and thrombotic signaling. Both RAS signaling and eicosanoid metabolism are critical to cardiovascular physiology and pathophysiology. However, the molecular interactions between RAS-related pathways and 20-HETE signaling in IHD remain incompletely understood^16^. Therefore, assessing ACE2 and 20-HETE simultaneously may provide valuable insights into the balance of vascular regulation in ACS, rather than viewing them as unrelated biomarkers. Identification of circulating biomarkers reflecting these molecular pathways may improve the understanding of disease mechanisms and facilitate early detection and risk stratification in patients with IHD.

This study aimed to investigate whether circulating ACE2 and 20-HETE are associated with ACS and may reflect dysregulation within a proposed ACE2/RAS–eicosanoid signaling framework, while also exploring the discriminatory performance and functional significance of 20-HETE.

## Methods

This cross-sectional translational study investigated whether dysregulation of the ACE2/RAS axis is associated with altered eicosanoid signaling in ACS and explored potential functional links using in vitro models.

### Study Population

A total of 122 patients with IHD that were hospitalized at the Department of Cardiology, Hospital of the Lithuanian University of Health Sciences (LUHS) Kauno Klinikos between 2023 and 2025 were included in this study. The study was conducted in accordance with the Declaration of Helsinki. Approval for the amendment of the biomedical research protocol was granted by the Kaunas Regional Biomedical Research Ethics Committee (Approval No. P1-BE-2-19/2019). All participants provided written informed consent.

Patient categorization was based on the guidelines of the European Society of Cardiology (ESC) for chronic coronary syndrome, ACS, and risk assessment using coronary imaging. The study participants were divided into two groups.

**Group I** (SCAD; *n* = 30). This group included 30 patients with exertional angina and clinically stable symptoms lasting ≥ 6 weeks without evidence of acute myocardial injury or electrocardiographic changes suggestive of ACS.

**Group II** (ACS; *n* = 92). This group comprised patients admitted to the hospital with a diagnosis of ACS, including ST-segment elevation myocardial infarction (STEMI), non-ST-segment elevation myocardial infarction (NSTEMI), or unstable angina, according to the criteria outlined in the ESC guidelines. All participants in this group underwent percutaneous coronary intervention (PCI) with implantation of a coronary stent. Patients received standard guideline-recommended pharmacological treatment.

**Exclusion criteria.** Patients with conditions affecting coagulation (e.g., malignancies, systemic inflammation), recent antibiotic therapy, renal insufficiency, hypothyroidism, hypovolemia, or refusal to participate were excluded.

All clinical, laboratory, and instrumental data were obtained from patients’ medical records. Venous blood samples for laboratory analyses, including biochemical testing and platelet function assessment, were collected according to a standardized protocol. In ACS patients, samples were obtained approximately 24 h after antiplatelet loading and before PCI, in accordance with ESC guideline-based care. In SCAD patients, samples were collected during planned clinical evaluation, under stable clinical conditions.

### Comparison group

The comparison group (**Group III**) was selected from the Kaunas, Lithuania cohort of the international Health, Alcohol and Psychosocial Factors in Eastern Europe (HAPIEE) study. Clinical and demographic information was obtained from the Kaunas HAPIEE baseline database collected in 2006–2008. Eligible participants were required to have no documented history of ischemic cardiovascular events, diabetes mellitus, or metabolic disorders in the available records. Initially, 69 individuals meeting these criteria were identified. After excluding 15 individuals who had died during follow-up before the present biomarker analysis in 2025 January, the final comparison group included 54 individuals with available clinical data and stored plasma samples. Plasma samples had been aliquoted and stored at − 80 °C until biomarker analysis.

### Experiments with cell cultures

The cell culture experiments were included as exploratory functional assays with the aim of providing biological plausibility for the clinical biomarker associations by assessing whether ACE2/RAS perturbation affects endothelial NO production, inflammatory gene expression, and CYP4F2/eicosanoid-related responses. Experiments were performed using three human cell lines: endothelial cells HUVEC sourced from Gibco (Life Technologies, Carlsbad, CA, USA), renal proximal tubule epithelial cells RPTEC/TERT1 (Evercyte, Vienna, Austria), and hepatic cells HepG2 (ATCC, Manassas, USA). HUVECs were cultured in Human Large Vessel Endothelial Cell Basal Medium containing Large Vessel Endothelial Supplement and 1% antibiotic solution (penicillin 10,000 IU/mL and streptomycin 10 mg/mL). RPTEC/TERT1 cells were maintained in DMEM/F-12 (1:1) medium supplemented with 4% fetal bovine serum, 2 mM L-alanyl-L-glutamine, 1× insulin-transferrin-selenium (ITS-G), 10 ng/mL human epidermal growth factor, 25 ng/mL hydrocortisone, and 100 µg/mL G418 sulfate. HepG2 cells were cultured in DMEM supplemented with 10% fetal bovine serum, 2 mM L-alanyl-L-glutamine, and 1% penicillin-streptomycin.

All cells were maintained at 37 °C in a humidified atmosphere with 5% CO_2_. Before experiments, cell viability and density were assessed. Cells were exposed to clinically relevant concentrations of captopril (0.25–2.25 µM; 98% purity, Sigma-Aldrich, St. Louis, USA), losartan (0.75–2.75 µM; 98% purity, Sigma-Aldrich, St. Louis, USA), and the ACE2 inhibitor MLN-4760 (0.05–1 µM; 99.8% purity, MedChemExpress, USA) for 24 h. Stock solutions were prepared in appropriate solvents (PBS or DMSO) and diluted in complete culture medium immediately before use. The selected concentration ranges of captopril and losartan were based on therapeutic plasma levels observed in clinical practice to ensure translational relevance.

Cellular metabolic activity was determined using the colorimetric 3-(4,5-dimethylthiazol-2-yl)-2,5-diphenyl-2 H-tetrazolium bromide (MTT) reduction assay. Cell cultures were incubated with MTT solution (5 mg/mL) for 3.5 h. Absorbance was measured at 550 nm with a 620 nm reference using an Infinite M Plex microplate reader (Tecan Austria GmbH, Grödig, Austria). Untreated cells served as controls (100% viability).

NO production was assessed in HUVEC culture supernatants using a colorimetric assay (Invitrogen, USA) that quantifies its stable metabolites, nitrite, and nitrate. Nitrate was enzymatically converted to nitrite, which was then detected using the Griess reaction. Absorbance was measured spectrophotometrically, and total NO levels were calculated from standard curves as the sum of nitrite and nitrate concentrations. Acetylcholine-treated cells were used as a positive control.

The quantitative real-time PCR (qPCR) was used to evaluate gene expression in accordance with MIQE guidelines. Total RNA was isolated, purified, and reverse transcribed into cDNA using standard protocols. qPCR was performed using SYBR Green chemistry (Applied Biosystems, USA), with gene-specific primers targeting inflammation-associated genes. Relative gene expression was calculated using the 2^-ΔΔCt^ method, with normalization to the reference gene TFRC. Results were expressed as fold changes relative to control samples.

### Genomic DNA extraction and genotyping by real-time PCR

Venous blood samples were collected into K2EDTA vacuum tubes. Genomic DNA was isolated from leukocytes using the salting-out method or a Genomic DNA Purification Kit (Thermo Fisher Scientific, Waltham, USA) according to the manufacturer’s instructions.

Genotyping of the *CYP4F2* gene variants (rs2108622, rs1558139, rs3093135, and rs2074902) was performed by real-time polymerase chain reaction (PCR) using TaqMan™ SNP Genotyping Assays. The PCR reaction mixture contained TaqMan^®^ Universal Master Mix II, TaqMan™ SNP Genotyping Assay, PCR-grade water, and genomic DNA (10 ng per reaction). Reactions were performed on 96-well plates, including control samples with known genotypes and a no-template control (NTC). PCR conditions consisted of an initial denaturation at 95 °C for 10 min, followed by 40 cycles of 95 °C for 15 s and 60 °C for 90 s. Amplification and allelic discrimination were performed using the QuantStudio 5 Real-Time PCR System (Thermo Fisher Scientific, Waltham, USA).

### Platelet aggregation assay

Platelet aggregation was assessed using the classical Born method with a Platelet Aggregometer TA-8 V (SD Innovation, Frouard, France). Venous blood was collected into vacuum tubes containing 3.2% sodium citrate and analyzed within 1 h of sampling. Samples were centrifuged to obtain platelet-rich plasma (PRP) and platelet-poor plasma (PPP). PRP was used for aggregation measurements, whereas PPP served as the reference sample for calibration of baseline light transmission.

Platelet aggregation was induced by the addition of adenosine diphosphate (ADP) and epinephrine as agonists. Changes in light transmission, reflecting platelet aggregation, were recorded for 10 min using the aggregometer. As platelets formed aggregates, light transmission increased.

### Extraction of eicosanoids from plasma samples

20-HETE was isolated and purified from plasma samples obtained from 176 study participants using a liquid-liquid extraction procedure. To prevent oxidation, samples were stabilized with butylated hydroxytoluene (10 mg/mL) and subsequently acidified to pH 4 using acetic acid (≥ 99.5%). Eicosanoids were then extracted with ethyl acetate at a 1:1 (v/v) ratio. The mixture was vigorously vortexed and centrifuged at 2500 × g for 20 min to achieve phase separation. The upper organic phase was carefully collected and evaporated under a nitrogen stream at 40 °C using a Multivap evaporator (Organomation, Berlin, USA). The dried residue was re-dissolved in 96% ethanol and further diluted with the sample dilution buffer before ELISA measurement.

### Determination of 20-HETE

Quantitative determination of 20-HETE in eicosanoid extracts obtained from blood plasma samples was performed using a competitive in vitro 20-HETE ELISA kit (Detroit R&D, Inc., Detroit, USA) following the manufacturer’s instructions. According to the manufacturer’s specifications, no significant cross-reactivity or interference has been observed between 20-HETE and structurally related fatty acids, including arachidonic, linoleic, and linolenic acids. Optical density was measured at 450 nm using an Infinite M Plex microplate reader.

### Determination of ACE2

Plasma ACE2 concentrations were measured using a sandwich-type ACE2 ELISA kit (BioVendor, Brno, Czech Republic). ACE2 was detected using a biotin-conjugated monoclonal anti-ACE2 antibody. The enzymatic reaction was developed using the substrate 3,3’,5,5’-tetramethylbenzidine. Optical density was measured at 450 nm using an Infinite M Plex microplate reader. According to the manufacturer’s specifications, the intra-assay coefficient of variation (CV) was 3.2%, while the inter-assay CV was 3.6%.

### Statistical data analysis

Quantitative variables were analyzed using the Kruskal–Wallis test. When the overall test was significant, post hoc pairwise comparisons were performed using the Mann–Whitney U test with Bonferroni correction. Categorical variables were compared using the χ^2^ test or Fisher’s exact test, as appropriate. Post hoc pairwise comparisons for categorical variables were performed using Fisher’s exact test with Bonferroni correction. The conformity of genotype distributions with the Hardy–Weinberg equilibrium within each group was tested using the χ^2^ test. The discriminative and prognostic performance of biomarkers was assessed using receiver operating characteristic (ROC) curve analysis. The area under the ROC curve (AUC) with 95% confidence interval (CI) was calculated, and the optimal cut-off value was determined using the Youden index (J = sensitivity + specificity − 1). Additional sensitivity logistic regression models were performed to assess whether the associations of circulating 20-HETE and ACE2 with ACS were influenced by clinically relevant covariates. The biomarkers were evaluated in separate models adjusted for age and sex, body mass index (BMI), smoking status, arterial hypertension, angiotensin-converting enzyme inhibitor (ACEI)/angiotensin receptor blocker (ARB) use, statin use, antiplatelet therapy, creatinine level, leukocyte count, and CRP level.

## Results

### Study population

A total of 176 participants were included: 54 healthy controls, 30 with SCAD, and 92 with ACS. There was a significant difference in age between the groups (*p* < 0.001), but BMI was similar across all groups (Table 1). Patients with ACS had slightly lower diastolic blood pressure and higher heart rates than controls. There were differences in comorbidities and medication use between the groups (Table 2).

**Table 1:** Demographic, anthropometric, hemodynamic, and lifestyle characteristics.

Parameter	Comparison (*n* = 54)	SCAD (*n* = 30)	ACS (*n* = 92)	*p*
Men, n (%)	30 (55.6%)	23 (76.7%)	67 (72.8%)	0.053
Women, n (%)	24 (44.4%)	7 (23.3%)	25 (27.2%)	0.053
Age, years	71.00 (46.00–72.00)^a^	62.00 (48.00–80.00)^a^	66.46 (39.02–85.19)	**< 0.001**
Anthropometric and hemodynamic parameters, median (min–max)				
Height, cm	164.25 (144.00–177.50)^b, c^	172.50 (156.00–195.00)^b^	172.00 (150.00–195.00)^c^	**< 0.001**
Weight, kg	77.25 (54.00–122.00)^d, e^	88.00 (60.00–123.00)^d^	86.00 (55.00–164.00)^e^	**< 0.001**
BMI, kg/m²	28.90 (21.42–42.46)	28.40 (21.26–38.70)	29.04 (19.72–43.13)	0.956
Systolic BP, mmHg	135.33 (107.00–180.67)	133.50 (102.00–189.00)	135.50 (97.00–200.00)	0.690
Diastolic BP, mmHg	84.67 (61.00–108.67)^f^	83.00 (65.00–110.00)	80.00 (52.00–128.00)^f^	**0.01**
Heart rate, beats/min	64.50 (44.00–99.00)^g^	68.50 (47.00–115.00)	70.00 (44.00–160.00)^g^	**0.01**
Smoking, n (%)				
Current smoker	1 (1.9%)^h, i^	9 (30.0%)^h^	23 (25.0%)^i^	**< 0.001**
Former smoker	0 (0.0%)	7 (23.3%)	0 (0.0%)	**< 0.001**
Never smoked	53 (98.1%)	13 (43.3%)	67 (72.8%)	**< 0.001**

Data are presented as median (min–max) or *n* (%). *p-*values were obtained using the Kruskal–Wallis test (χ^2^ test for categorical parameters), followed by Bonferroni-corrected Mann–Whitney U test for pairwise comparisons. Pairwise comparisons: a – *p* < 0.001; b, c, e, h, i – *p* = 0.003; d – *p* = 0.033; f – *p* = 0.009; g – *p* = 0.006.

ACS – acute coronary syndrome; BMI – body mass index; BP – blood pressure; SCAD – stable coronary artery disease.

**Table 2:** Comorbidities and medication use in the study population.

Parameter	Comparison (*n* = 54)	SCAD (*n* = 30)	ACS (*n* = 92)	*p*
Arterial hypertension	41 (75.9%)^**a**^	28 (93.3%)^**b**^	34 (37.0%)^**a, b**^	**< 0.001**
Diabetes mellitus	0 (0.0%)	6 (20.0%)	30 (32.6%)	**< 0.001**
History of MI	0 (0.0%)	0 (0.0%)	26 (28.3%)	**< 0.001**
ACEI use	0 (0.0%)	18 (60.0%)^**c**^	90 (97.8%)^**c**^	**< 0.001**
ARB use	0 (0.0%)	8 (26.7%)^**d**^	2 (2.2%)^**d**^	**< 0.001**
Statin use	2 (3.7%)^**e, f**^	27 (90.0%)^**e**^	90 (97.8%)^**f**^	**< 0.001**
Aspirin use	0 (0.0%)	18 (60.0%)^**g**^	82 (89.1%)^**g**^	**< 0.001**
Clopidogrel use	0 (0.0%)	13 (43.3%)	28 (30.4%)	**< 0.001**
Ticagrelor use	0 (0.0%)	0 (0.0%)	59 (64.1%)	**< 0.001**

Data are presented as *n* (%). *p-*values were calculated using Fisher’s exact test. Pairwise comparisons were performed with Bonferroni correction: a–g – *p* < 0.003.

ACEI – angiotensin-converting enzyme inhibitors; ACS – acute coronary syndrome; ARB – angiotensin II receptor blockers; MI – myocardial infarction; SCAD – stable coronary artery disease.

### Laboratory characteristics

Among laboratory markers (Table 3), ACS patients had higher leukocyte counts, creatinine levels, and AST activity than SCAD patients (all *p* ≤ 0.017).

**Table 3:** Biochemical and hematological blood parameters in the study population.

Parameter	Comparison (*n* = 54)	SCAD (*n* = 30)	ACS (*n* = 92)	*p*
Glucose, mmol/L	5.60 (4.30–7.00)	5.98 (4.70–7.30)	5.27 (5.27–5.27)	0,465
HDL cholesterol, mmol/L	1.45 (0.92–2.38)^**a**^	1.18 (0.85–1.66)^**a**^	1.22 (1.08–1.35)	**0**,**002**
LDL cholesterol, mmol/L	3.63 (1.14–6.32)^**b**^	2.75 (0.66–5.04)^**b**^	2.65 (2.39–2.90)	**0**,**012**
Triglycerides, mmol/L	1.15 (0.48–4.54)	1.58 (0.62–4.28)	1.12 (1.03–1.21)	0,062
Hemoglobin, g/L	ND	147.00 (126.00–173.00)	138.00 (94.00–165.00)	**0**,**002**
Leukocytes, ×10^9^/L	ND	6.92 (3.98–15.80)	8.47 (2.93–17.15)	**0**,**017**
Platelets, ×10^9^/L	ND	235.00 (121.00–371.00)	216.00 (115.00–552.00)	0,107
Creatinine, µmol/L	ND	74.75 (55.00–120.00)	94.00 (56.00–270.00)	**< 0**,**001**
AST, U/L	ND	15.50 (2.00–64.37)	31.00 (1.00–576.00)	**0**,**004**
ALT, U/L	ND	29.00 (9.00–57.00)	29.00 (2.00–138.00)	0,418
INR	ND	0.99 (0.97–1.10)	1.10 (0.91–2.53)	0,478
CRP, mg/L	ND	3.92 (0.50–7.00)	2.62 (1.00–177.90)	0,370

Data are presented as median (min–max). *p*-values were calculated using the Kruskal–Wallis test, followed by the Bonferroni-corrected Mann–Whitney U test for pairwise comparisons. Pairwise comparisons: a – *p* = 0.003; b – *p* = 0.03. ACS – acute coronary syndrome; ALT – alanine aminotransferase; AST – aspartate aminotransferase; CRP – C-reactive protein; HDL – high-density lipoprotein; INR – international normalized ratio; LDL – low-density lipoprotein; ND – not determined; SCAD – stable coronary artery disease.

### Genetic analysis

Genotype distributions of *CYP4F2* polymorphisms in the comparison group were consistent with Hardy–Weinberg equilibrium: rs2108622 (*p* = 0.389), rs1558139 (*p* = 0.380), rs3093135 (*p* = 0.319), and rs2074902 (*p* = 0.235). Genotype distributions of *CYP4F2* polymorphisms (rs2108622, rs1558139, rs3093135, rs2074902) did not differ significantly between study groups (Supplemental Table 1).

### Circulating ACE2 and 20‑HETE

**Fig. 1:**
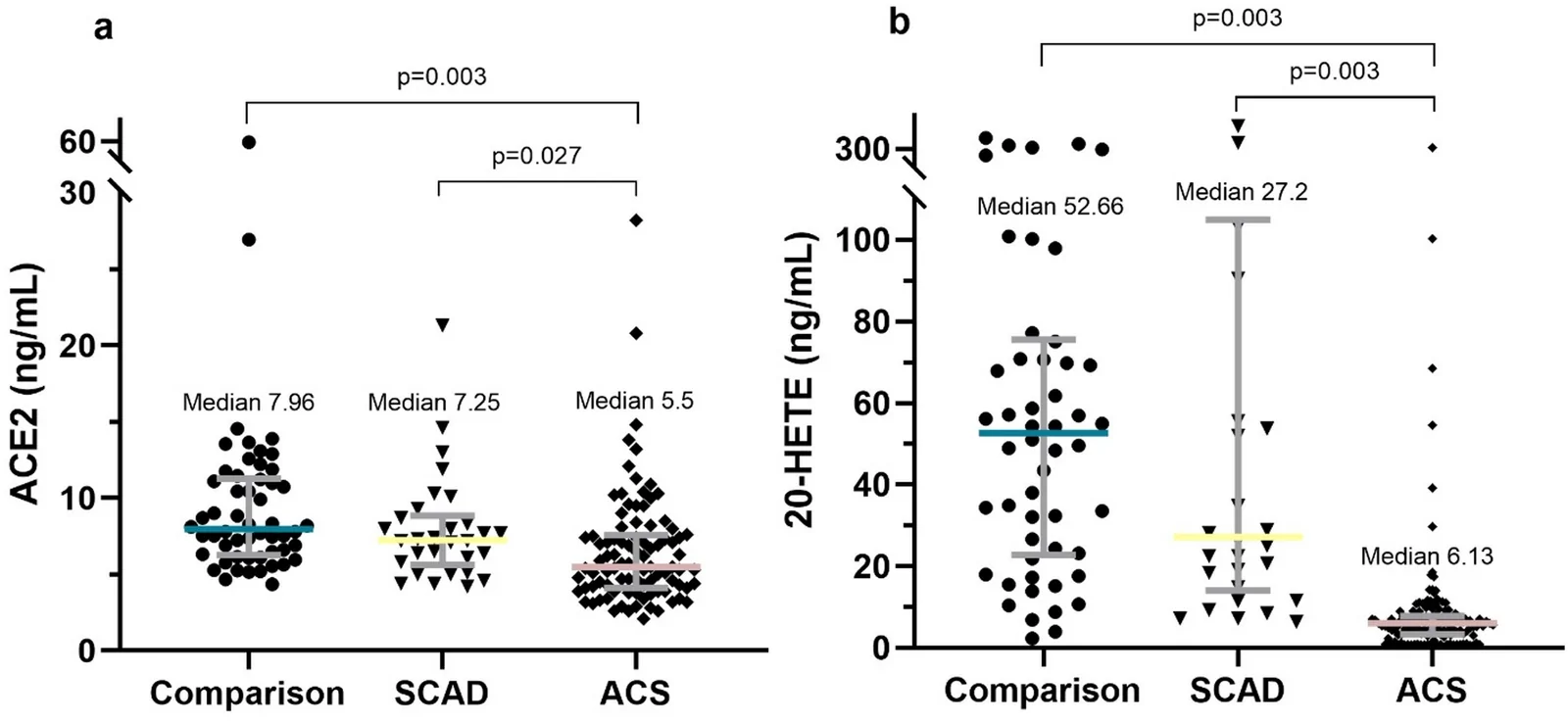
Distribution of circulating ACE2 and 20-HETE levels across the study groups. Individual data points are shown for ACE2 (**a**) and 20-HETE (**b**) concentrations in the comparison, SCAD, and ACS groups. Horizontal lines and error bars indicate medians with interquartile ranges. Differences between two groups were assessed using the Mann–Whitney U test with Bonferroni correction following an overall Kruskal–Wallis test. *p*-values shown above the plots indicate significant pairwise comparisons. Effect sizes are presented as median differences between groups: for ACE2, comparison vs. ACS Δmedian = 2.46 ng/mL and SCAD vs. ACS Δmedian = 1.75 ng/mL; for 20-HETE, comparison vs. ACS Δmedian = 46.53 ng/mL and SCAD vs. ACS Δmedian = 21.07 ng/mL. 20-HETE – 20-hydroxyeicosatetraenoic acid; ACE2 – angiotensin-converting enzyme 2.

Plasma ACE2 and 20-HETE levels (Fig. 1) showed significant differences between the study groups (both *p* < 0.001). The median ACE2 concentrations were 7.96 ng/mL in healthy controls, 7.25 ng/mL in SCAD, and 5.50 ng/mL in ACS. Patients with ACS had notably lower ACE2 levels compared to controls (*p* < 0.001) and SCAD patients (*p* = 0.009). A clear decreasing trend in 20-HETE concentrations was seen across groups: 52.66 ng/mL in healthy controls, 27.20 ng/mL in SCAD, and 6.13 ng/mL in ACS (*p* < 0.001). ACS patients exhibited significantly lower 20-HETE levels than both SCAD and healthy controls.

### Diagnostic performance of 20‑HETE

In multivariable logistic regression analysis (Table 4), both 20-HETE and ACE2 were independently associated with ACS. However, adding ACE2 to 20-HETE did not materially improve diagnostic discrimination, as the combined model showed AUC values similar to those of 20-HETE alone (Table 5). Sensitivity logistic regression models showed that the inverse association between circulating 20-HETE and ACS remained largely consistent after adjustment for most clinically relevant covariates. In contrast, the association between ACE2 and ACS was attenuated after adjustment for ACEI/ARB use, antiplatelet therapy, creatinine level, leukocyte count, and CRP level (Supplemental Table 2).

**Table 4:** Multivariable logistic regression analysis for ACS prediction.

Variable	OR	95% CI	*p*
20-HETE (ng/mL)	0.953	0.934–0.972	**< 0**,**001**
ACE2 (ng/mL)	0.884	0.796–0.982	**0**,**022**

20-HETE – 20-hydroxyeicosatetraenoic acid; ACE2 – angiotensin-converting enzyme 2; CI – confidence interval; OR – odds ratio.

**Table 5:** The effects of 20-HETE alone and in combination with ACE2 on diagnostic performance.

Groups	AUC	95% CI	Cut-off value	Sensitivity %	Specificity %
Combined 20-HETE and ACE2					
ACS vs. SCAD	0.87	0.79–0.94	-	84.8	80
ACS vs. comparison	0.93	0.88–0.97	-	90.2	88.9
ACS vs. non-ACS (comparison + SCAD)	0.91	0.86–0.96	-	89.1	82.7
20-HETE alone					
ACS vs. SCAD	0.91	0.84–0.97	≤ 11.34 ng/mL	86.9	83.3
ACS vs. comparison	0.93	0.88–0.97	≤ 13.55 ng/mL	90.2	88.9
ACS vs. non-ACS (comparison + SCAD)	0.92	0.87–0.96	≤ 13.55 ng/mL	90.2	84.5

20-HETE – 20-hydroxyeicosatetraenoic acid; ACE2 – angiotensin-converting enzyme 2; ACS – acute coronary syndrome; AUC – area under the curve; CI – confidence interval; SCAD – stable coronary artery disease.

ROC analysis showed that circulating 20-HETE levels effectively distinguish ACS from SCAD (Supplemental Fig. 1a), with an AUC of 0.91. The best cutoff point, determined by the Youden index, was ≤ 11.34 ng/mL, yielding 86.9% sensitivity and 83.3% specificity. Overall, these results indicate that lower circulating 20-HETE concentrations are associated with acute coronary events and show discriminatory performance in the present cohort. Supplemental Figs. 1b and 1c illustrate the ROC curves for discrimination between ACS and the comparison group, and between ACS and the combined non-ACS group (comparison + SCAD).

### Exploratory cell culture findings

In HUVEC cells, pharmacological modulation of ACE2/RAS signaling altered NO production. Captopril caused a strong and steady increase in NO levels, raising them from 8.7 (7.0–11.3) µM in control cells to 20.0–24.5 µM after treatment (Supplemental Table 3). The highest NO levels were observed at 2.25 µM. Losartan had a weaker, more dose-dependent effect, with significant NO increases only at 2.25 and 2.75 µM. On the other hand, the ACE2 inhibitor MLN-4760 lowered NO production, and at 1 µM, NO levels dropped from 11.3 (9.6–13.6) to 6.3 (5.3–8.6) µM.

Captopril strongly reduced the expression levels of both adhesion molecule genes. *VCAM-1* dropped to about 0.3 times the control level, and *ICAM-1* fell to about 0.2 times at higher doses (Supplemental Table 4). This pattern was seen across all tested concentrations. Losartan did not change *VCAM-1* or *ICAM-1* much, as both stayed near control levels. In contrast, MLN-4760 increased *ICAM-1* by 1.3- to 1.7-fold relative to control, while *VCAM-1* remained about the same.

*CYP4F2* levels varied with treatment (Supplemental Table 4). Captopril caused a small increase in *CYP4F2* only at higher doses, up to about 1.3 times the control. Losartan, on the other hand, reduced *CYP4F2* at all doses, down to 0.2–0.3 times the control value. In contrast, MLN-4760 increased *CYP4F2* expression from 1.4 times at 0.5 µM to 2.0 times at 1 µM.

In HUVEC, RPTEC/TERT1, and HepG2 cells, 20-HETE reduced cellular metabolic activity in a concentration-dependent manner (Supplemental Table 5). In HUVEC cells, activity dropped from 94.3% of control at 1 nM to 80.7% at 100 nM. In RPTEC/TERT1 cells, the decrease started at 2 nM and reached 79.0% at 100 nM. In HepG2 cells, the effect was strongest, with activity falling to 74.8% at 100 nM, even at the lowest doses. At the same time, 20-HETE raised G protein-coupled receptor 75 (GPR75) levels in HUVEC and HepG2 cells at all concentrations, and from 10 nM upward in RPTEC/TERT1 cells.

## Discussion

This study demonstrates that reduced circulating ACE2 and 20-HETE levels are associated with ACS and supports a hypothesis-generating model of dysregulation within the conceptual ACE2/RAS–eicosanoid signaling network linked to endothelial dysfunction. Using clinical biomarker profiling alongside exploratory cell-based experiments, our findings suggest that lower circulating 20-HETE levels are accompanied by coordinated changes in ACE2-related vascular signaling, NO availability, inflammatory response, CYP4F2-associated eicosanoid regulation, and 20-HETE–GPR75 signaling. Together, these results support a conceptual model in which disruption of the protective ACE2/RAS balance is associated with altered 20-HETE signaling within a broader endothelial stress response, potentially contributing to the instability seen in acute coronary cases. We propose this hypothesis-generating ACE2/RAS-eicosanoid framework for interpreting the clinical findings, while recognizing that the cross-sectional clinical design cannot establish causality or temporal direction.

A major clinical observation was the marked reduction of circulating 20-HETE in ACS compared with SCAD and comparison groups. Patients with ACS exhibited notably lower 20-HETE levels (6.13 ng/mL) compared to both SCAD patients (27.20 ng/mL) and healthy controls (52.66 ng/mL), and 20-HETE effectively differentiated ACS from SCAD with an AUC of 0.91, using a threshold of ≤ 11.34 ng/mL that achieved 87% sensitivity and 83% specificity. However, this result should be interpreted as evidence of association and discrimination in the present cohort rather than as proof of a standalone diagnostic utility. External validation and direct comparison with established clinical markers are required before clinical implementation can be considered. The lower 20-HETE levels observed during ACS may reflect altered synthesis, increased local consumption, redistribution, or degradation of this lipid mediator during acute endothelial stress, inflammation, and thrombosis.

These findings are supported by previous studies showing that lipid mediator dynamics change rapidly during ischemia and may precede conventional markers of myocardial necrosis^17–19^. Furthermore, lower ACE2 levels observed in our ACS group (5.50 ng/mL) are consistent with findings from heart failure and acute ischemia registries, where reduced ACE2 activity has been associated with higher Killip class and worse short-term outcomes^11,20,21^. Although our findings compare favorably with traditional markers of vascular inflammation and plaque instability, such as hs-CRP and MPO, which typically show lower AUC values (≈ 0.65–0.75) when distinguishing ACS from SCAD^22,23^ they should not be interpreted as evidence that 20-HETE can replace established biomarkers. Cardiac troponins (hs-cTn) remain the gold standard for detecting myocardial necrosis, whereas 20-HETE may reflect a different pathophysiological process^24,25^. Therefore, 20-HETE should not be interpreted as a replacement for established biomarkers such as hs-cTn or inflammatory markers, but rather as a candidate complementary biomarker that may provide information on vascular and eicosanoid pathway dysregulation in ACS.

The development of ACS involves complex interactions among endothelial dysfunction, vascular inflammation, oxidative stress, and thrombosis^26–28^. Both the RAS and arachidonic acid-derived lipid mediators are central to the regulation of these physiological processes. ACE2 is a key counter-regulatory enzyme within the RAS that converts angiotensin II to angiotensin-(1–7), thereby exerting vasodilatory, anti-inflammatory, and cardioprotective effects^29,30^. Reduced ACE2 activity has been associated with endothelial dysfunction and adverse cardiovascular outcomes, partly through impaired angiotensin II degradation, coronary microvascular dysfunction, and systemic inflammation^31,32^. In our study, 97.8% of patients with ACS received ACEI. Although ACEI reduce the formation of angiotensin II, they do not directly increase ACE2 levels. Severe RAS dysregulation during ischemia may therefore exceed the compensatory capacity of the ACE2/Ang-(1–7) axis^30,33^. Consistent with this, we observed a significant decline in ACE2 levels in ACS patients. This suggests a potential suppression of the protective RAS axis, which has been linked to adverse myocardial remodeling and increased systemic inflammatory injury^11^.

The in vitro experiments were designed to provide functional context for the clinical biomarker findings rather than to reproduce ACS in its full systemic complexity. Therefore, we focused on selected readouts relevant to the proposed ACE2/RAS–eicosanoid framework, including NO production as an indicator of endothelial function, *ICAM-1*/*VCAM-1* expression as markers of inflammatory endothelial activation, *CYP4F2* expression as a link to 20-HETE biosynthesis, and *GPR75* expression as a downstream 20-HETE-related signaling readout. Pharmacological modulation of the ACE2/RAS axis affected NO production in endothelial cells. Captopril, and to a lesser degree losartan, supported endothelial-protective responses, while blocking ACE2 weakened a key protective mechanism. Lower ACE2 activity was associated with lower NO levels, consistent with signs of endothelial dysfunction. Overall, these results suggest that captopril creates an anti-inflammatory and stabilizing effect on endothelial cells by lowering molecules involved in white blood cell adhesion and activation. In contrast, blocking ACE2 made the cells more pro-inflammatory, shown by higher *ICAM-1* levels. Thus, reduced ACE2 activity appears to be linked to both lower NO levels and broader inflammatory changes in endothelial cells.

Alongside RAS signaling, metabolites derived from arachidonic acid have been identified as key regulators of vascular homeostasis. Among these, 20-HETE is particularly significant in regulating vascular tone and endothelial function^15,34^. Experimental studies indicate that 20-HETE contributes to endothelial dysfunction by activating NADPH oxidase, increasing reactive oxygen species production, and initiating downstream inflammatory signaling^35–37^. However, the marked reduction in circulating 20-HETE in ACS appears paradoxical, given the pro-inflammatory and vasoconstrictive effects of 20-HETE in other settings. Several non-mutually exclusive explanations are possible. First, circulating 20-HETE may decrease because of increased local tissue uptake or consumption during acute thrombo-inflammatory activation. Second, acute oxidative stress may impair CYP450 activity, thereby reducing systemic 20-HETE synthesis when vascular signaling is disrupted [38,39]. Under physiological conditions, 20-HETE is synthesized from arachidonic acid by cytochrome P450 enzymes (CYP4A/4F) and functions as an intracellular second messenger^15^. However, during myocardial ischemia, increased oxidative stress and activation of eicosanoid pathways may impair CYP450 activity. High levels of reactive oxygen species can inactivate CYP450 enzymes, potentially reducing 20-HETE synthesis when its regulatory role is most critical^38,39^. Third, plasma 20-HETE may not fully reflect compartment-specific intracellular or vascular-wall 20-HETE signaling. Therefore, low circulating 20-HETE should not be interpreted as evidence that 20-HETE is absent or biologically inactive during ACS; rather, it may indicate altered distribution or regulation of the eicosanoid pathway during acute ischemia. The cell culture findings support this cautious interpretation by showing that the ACE2/RAS axis is linked to eicosanoid metabolism, but the connection is not one-way. Instead of a simple model in which lower ACE2 levels always correlate with lower CYP4F2 activity, the data suggest that different ways of altering RAS affect CYP4F2 activity differently. Therefore, changes in ACE2 and 20-HETE should be seen as signs of broader dysregulation in the RAS–eicosanoid system. These results support the concept that 20-HETE is not just a biomarker but an active lipid mediator. In addition, 20-HETE exposure reduced cellular metabolic activity, which may signal stress or reduced cell health, and increased *GPR75* levels, showing activation of a 20-HETE-related signaling pathway. This supports the idea that the 20-HETE system may play a direct role in how cells respond to acute injury. Taken together, our data support a conceptual model in which dysregulation of the ACE2/RAS axis is associated with reduced NO bioavailability and endothelial activation, accompanied by altered CYP4F2-dependent eicosanoid metabolism and 20-HETE–GPR75 signaling. So, changes in ACE2 and 20-HETE should be seen as part of a connected ACE2/RAS–NO–inflammation–CYP4F2/20-HETE–GPR75 system, not as separate problems.

Genetic analysis revealed that *CYP4F2* polymorphisms (rs2108622, rs1558139, rs3093135, rs2074902) did not explain the differences in circulating 20-HETE between groups. This suggests that the metabolic response to acute myocardial ischemia may outweigh genetic influences on lipid mediator synthesis^40^. However, because only selected variants were analyzed, broader genetic or epigenetic influences on 20-HETE metabolism cannot be excluded.

From a clinical perspective, the role of 20-HETE in cardiovascular disease is complex. Previous studies demonstrated that 20-HETE exerts diverse and sometimes opposing effects on vascular function^15^. 20-HETE acts as a potent vasoconstrictor and proinflammatory mediator that promotes endothelial dysfunction and oxidative stress through NADPH oxidase activation^41^. These properties traditionally link elevated 20-HETE levels to the progression of hypertension and atherosclerosis. On the other hand, 20-HETE participates in regulatory pathways involved in natriuresis and vascular remodeling, suggesting that its biological effects depend on the clinical context and the stage of IHD^42^. In our study, patients with ACS exhibited slightly lower diastolic blood pressure compared to controls. In patients with coronary artery disease, low diastolic blood pressure is associated with increased risk of myocardial infarction^43^. The effect of 20-HETE on blood pressure is complex because, despite its vasoconstrictor actions, it can also reduce blood pressure by inhibiting sodium transport in the kidney^44^. Other systemic factors, including nutritional status, may also contribute to cardiovascular risk and biomarker variability^45^.

By distinguishing ACS from SCAD, circulating 20-HETE may capture aspects of vascular instability associated with the transition from stable atherosclerosis to acute coronary events^46^. Measurement of circulating 20-HETE and ACE2 may provide additional insights into vascular regulatory balance that conventional necrosis markers do not capture. Cardiac troponins primarily indicate myocardial injury following necrosis, whereas changes in lipid mediators such as 20-HETE may signal earlier pathophysiological processes, including endothelial dysfunction, vascular inflammation, and disrupted signaling pathways^19,47^. Simultaneous assessment of ACE2 and 20-HETE could enhance understanding of the interplay between protective RAS signaling and eicosanoid-mediated vascular regulation. Individuals exhibiting low concentrations of both biomarkers may constitute a subgroup with compromised vascular protective mechanisms, indicating disruption of both ACE2-dependent anti-inflammatory signaling and CYP450-derived lipid mediator pathways^10,48^. The results underscore the potential of circulating lipid mediators to serve as indicators of vascular instability that may precede acute coronary events. However, clinical utility will require external validation, standardized pre-analytical procedures, and testing of incremental value beyond hs-cTn, hs-CRP, renal function, medication exposure, and conventional clinical risk factors. Individuals with low concentrations of both biomarkers may represent a subgroup with altered vascular protective signaling, but this subgroup hypothesis should be tested prospectively.

**Fig. 2:**
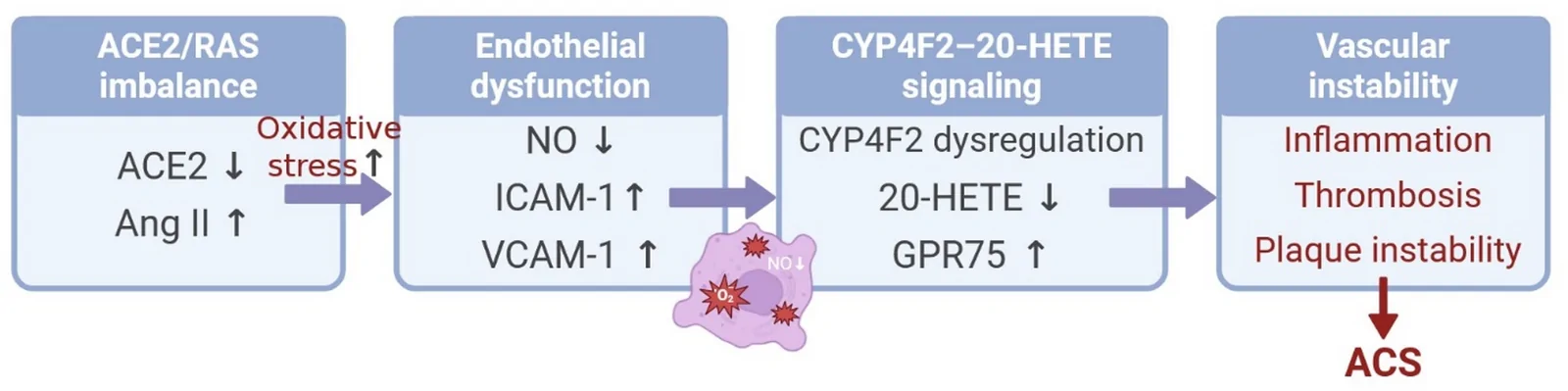
Proposed model of ACE2/RAS–eicosanoid axis dysregulation in ACS. The schematic illustrates how dysregulation of the ACE2/RAS–eicosanoid signaling pathway contributes to ACS. A decline in ACE2 activity skews the RAS toward a pro-inflammatory and vasoconstrictive state, reducing NO availability and causing endothelial dysfunction. This imbalance leads to endothelial activation, with increased expression of adhesion molecules like ICAM-1 and VCAM-1. Additionally, changes in RAS signaling are linked to disrupted CYP4F2-dependent eicosanoid metabolism. In ACS, circulating 20-HETE levels are significantly decreased, possibly due to increased consumption or defective synthesis during acute inflammation and clot formation. At the cellular level, 20-HETE influences metabolic activity and activates GPR75 signaling, suggesting its role as an active lipid mediator rather than just a marker. Overall, these mechanisms form an interconnected ACE2/RAS–NO–inflammation–CYP4F2–20-HETE–GPR75 signaling network that links endothelial dysfunction to vascular instability and the onset of acute coronary events.

This hypothesis-generating model is summarized in Fig. 2.

### Study limitations

Several limitations should be acknowledged. The relatively small, single-center cohort may restrict the generalizability of the proposed 20-HETE cut-off value, necessitating validation in larger, multicenter populations. No external validation cohort was available, and the incremental diagnostic value of 20-HETE beyond established biomarkers and clinical variables, including hs-cTn, hs-CRP, MPO, clinical risk scores, and renal function, was not directly tested. The cross-sectional design prevented evaluation of temporal changes in circulating 20-HETE and ACE2 levels, as well as their long-term prognostic significance for major adverse cardiovascular events. Age differences between study groups may have affected eicosanoid metabolism. Nutritional and inflammatory status may influence cardiovascular risk and biomarker levels. The comparison group was selected from the HAPIEE cohort, and differences in recruitment period, sample storage duration, and baseline characteristics may have affected biomarker distributions. Although *CYP4F2* genotyping was limited to selected variants, unmeasured genetic or epigenetic factors may still influence 20-HETE metabolism. Finally, although our in vitro experiments provide functional support for a biological interaction between the ACE2/RAS axis and eicosanoid signaling, they do not establish a direct causal mechanism linking reductions in circulating ACE2 and 20-HETE in patients with ACS. Future studies should therefore include direct assessment of oxidative stress pathways, endothelial vasoreactivity, and thrombosis-related functional endpoints to further clarify the mechanistic role of the proposed ACE2/RAS–eicosanoid axis in ACS.

## Supplementary Information

Below is the link to the electronic supplementary material.

Supplementary Material 1

## Data Availability

Data available from corresponding author upon reasonable request.

## Abbreviations

ACE2: Angiotensin-converting enzyme 2
ACEI: Angiotensin-converting enzyme inhibitors
ACS: Acute coronary syndrome
ADP: Adenosine diphosphate
AUC: Area under the ROC curve
CV: Coefficient of variation
CI: Confidence interval
GPR75: G protein-coupled receptor 75
HAPIEE: Health, alcohol and psychosocial factors in Eastern Europe
hs-CRP: High-sensitivity C-reactive protein
hs-cTn: High-sensitivity cardiac troponin
20-HETE: 20-hydroxyeicosatetraenoic acid
IHD: Ischemic heart disease
MPO: Myeloperoxidase
NO: Nitric oxide
NSTEMI: Non-ST-segment elevation myocardial infarction
NTC: No-template control
OR: Odds ratio
PC: Percutaneous coronary intervention
PPP: Platelet-poor plasma
PRP: Platelet-rich plasma
RAS: Renin-angiotensin system
ROC: Receiver operating characteristic
SCAD: Stable coronary artery disease
STEMI: ST-segment elevation myocardial infarction
TMB: 3,3’,5,5’-tetramethylbenzidine
