# Supplementary material for: Reduced circulating 20-hydroxyeicosatetraenoic acid and ACE2 in acute coronary syndrome with exploratory cell-based observations
Source: Sci Rep. 2026 Jun 10;16:26462. doi: 10.1038/s41598-026-56316-9 (PMC13500614; doi:10.1038/s41598-026-56316-9)
Supplement: Supplementary file 1 [file 41598_2026_56316_MOESM1_ESM.docx]

**SUPPLEMENTAL MATERIAL**

**Reduced circulating 20-hydroxyeicosatetraenoic acid and ACE2 in acute coronary syndrome with exploratory cell-based observations**

Ieva Čiapienė, Aistė Ivanauskienė, Nora Kupstytė-Krištaponė, Vaiva Lesauskaitė, Robertas Pranevičius, Gintarė Šakalytė, Daiva Stanislovaitienė, Cynthia Sithole, and Vacis Tatarūnas^*^

*Corresponding author: Vacis Tatarūnas

**Supplemental Tables**

**Supplemental Table 1.** Distribution of *CYP4F*2 rs2108622, rs1558139, rs3093135, and rs2074902 genotypes in the study population

| **Genetic analysis** | | | |
| --- | --- | --- | --- |
| **Genotype** | **Comparison**  **(n = 54)** | **SCAD**  **(n = 30)** | **ACS**  **(n = 92)** |
| *CYP4F2* rs2108622 | | | |
| CC | 33 (66.0%) | 15 (50.0%) | 57 (62.0%) |
| CT | 14 (28.0%) | 13 (43.3%) | 29 (31.5%) |
| TT | 3 (6.0%) | 2 (6.7%) | 6 (6.5%) |
| *CYP4F2* rs1558139 | | | |
| AA | 8 (16.0%) | 6 (20.0%) | 18 (19.6%) |
| AG | 30 (60.0%) | 14 (46.7%) | 50 (54.3%) |
| GG | 12 (24.0%) | 10 (33.3%) | 24 (26.1%) |
| *CYP4F2* rs3093135 | | | |
| AA | 34 (68.0%) | 16 (53.3%) | 55 (59.8%) |
| AT | 16 (32.0%) | 13 (43.3%) | 30 (32.6%) |
| TT | 0 (0.0%) | 1 (3.3%) | 7 (7.6%) |
| *CYP4F2* rs2074902 | | | |
| CC | 0 (0.0%) | 1 (3.3%) | 4 (4.3%) |
| CT | 16 (32.0%) | 12 (40.0%) | 22 (23.9%) |
| TT | 34 (68.0%) | 17 (56.7%) | 66 (71.7%) |

Data are presented as *n* (%).

Abbreviations: ACS – acute coronary syndrome; SCAD – stable coronary artery disease.

**Supplemental Table 2.** Sensitivity logistic regression models for the associations of circulating 20-HETE and ACE2 with ACS

| **Model** | **Adjustment variables** | **20-HETE OR** | **95% CI** | ***p*** | **ACE2 OR** | **95% CI** | ***p*** |
| --- | --- | --- | --- | --- | --- | --- | --- |
| Base model | None | 0.948 | 0.929–0.967 | **<0.001** | 0.849 | 0.768–0.937 | **0.001** |
| Model 1 | Age, sex | 0.948 | 0.928–0.967 | **<0.001** | 0.843 | 0.761–0.935 | **0.001** |
| Model 2 | BMI | 0.949 | 0.930–0.968 | **<0.001** | 0.841 | 0.758–0.935 | **0.001** |
| Model 3 | Smoking | 0.946 | 0.925–0.967 | **<0.001** | 0.849 | 0.767–0.940 | **0.002** |
| Model 4 | Arterial hypertension | 0.957 | 0.938–0.976 | **<0.001** | 0.855 | 0.772–0.947 | **0.003** |
| Model 5 | ACEI, ARB use | 0.976 | 0.958–0.994 | **0.009** | 0.894 | 0.788–1.016 | 0.085 |
| Model 6 | Statin use | 0.948 | 0.929–0.967 | **<0.001** | 0.848 | 0.767–0.937 | **0.001** |
| Model 7 | Aspirin, clopidogrel, ticagrelor use | 0.992 | 0.980–1.004 | 0.193 | 0.983 | 0.871–1.110 | 0.779 |
| Model 8 | Creatinine level | 0.981 | 0.963–0.999 | **0.042** | 0.907 | 0.804–1.022 | 0.109 |
| Model 9 | Leukocyte count, CRP level | 0.832 | 0.741–0.933 | **0.002** | 0.935 | 0.810–1.078 | 0.354 |

Data are presented as odds ratios (ORs) with 95% confidence intervals (CIs) and *p*-values. Circulating 20-HETE and ACE2 were evaluated in separate logistic regression models using the same listed adjustment variables.

Abbreviations: ACE2 – angiotensin-converting enzyme 2; ACEI – angiotensin-converting enzyme inhibitors; ACS – acute coronary syndrome; ARB – angiotensin receptor blockers; BMI – body mass index; CI – confidence interval; CRP – C-reactive protein; OR – odds ratio; 20-HETE – 20-hydroxyeicosatetraenoic acid.

**Supplemental Table 3.** Effects of ACE2/RAS modulation on endothelial NO production in HUVEC cells

| **RAS inhibitor** | **Concentration** | **NO concentration (µM)** | ***p*** |
| --- | --- | --- | --- |
| Captopril | 0 µM | 8.7 (7.0–11.3) | - |
|  | 0.25 µM | 20.0 (19.4–23.4) | **0.029** |
|  | 0.75 µM | 20.6 (18.8–27.1) | **0.029** |
|  | 1.25 µM | 21.4 (20.9–27.7) | **0.029** |
|  | 1.75 µM | 21.3 (20.1–27.9) | **0.029** |
|  | 2.25 µM | 24.5 (23.5–28.3) | **0.029** |
| Losartan | 0 µM | 7.9 (5.2–12.4) | - |
|  | 0.75 µM | 7.1 (3.5–10.3) | 0.686 |
|  | 1.25 µM | 7.9 (7.2–13.5) | 0.686 |
|  | 1.75 µM | 13.0 (10.2–14.2) | 0.057 |
|  | 2.25 µM | 16.4 (12.5–21.7) | **0.029** |
|  | 2.75 µM | 18.3 (14.5–23.7) | **0.029** |
| MLN-4760 | 0 µM | 11.3 (9.6–13.6) | - |
|  | 0.05 µM | 10.9 (9.6–13.6) | 0.886 |
|  | 0.1 µM | 10.1 (9.0–15.3) | 0.686 |
|  | 0.25 µM | 9.8 (8.0–12.6) | 0.486 |
|  | 0.5 µM | 7.4 (5.4–10.5) | 0.114 |
|  | 1 µM | 6.3 (5.3–8.6) | **0.029** |

Data are presented as median (min–max). *p-*values were calculated using the Mann–Whitney U test, comparing each concentration with the control group (cells incubated with medium without RAS inhibitors), based on *n* = 4 independent experiments.

Abbreviations: NO – nitric oxide; RAS – renin-angiotensin system.

**Supplemental Table 4.** Effects of ACE2/RAS modulation on endothelial inflammatory gene expression in HUVEC cells

| **RAS inhibitor** | **Concentration** | ***VCAM-1***  **expression (FC)** | ***ICAM-1* expression (FC)** | ***CYP4F2***  **expression (FC)** |
| --- | --- | --- | --- | --- |
| Captopril | 0.25 µM | 0.4 (0.3–0.5)* | 0.4 (0.3–0.4)* | 1.0 (0.7–1.1) |
|  | 0.75 µM | 0.4 (0.3–0.5)* | 0.3 (0.3–0.4)* | 1.1 (1.0–1.3) |
|  | 1.25 µM | 0.3 (0.3–0.4)* | 0.3 (0.2–0.4)* | 1.1 (1.0–1.2) |
|  | 1.75 µM | 0.3 (0.3–0.4)* | 0.2 (0.2–0.3)* | 1.3 (1.1–1.6)* |
|  | 2.25 µM | 0.3 (0.2–0.3)* | 0.2 (0.2–0.3)* | 1.3 (1.2–1.7)* |
| Losartan | 0.75 µM | 0.9 (0.5–1.2) | 1.1 (0.9–1.5) | 0.3 (0.1–0.7)* |
|  | 1.25 µM | 0.9 (0.6–1.3) | 1.0 (0.5–1.0) | 0.3 (0.1–0.4)* |
|  | 1.75 µM | 0.8 (0.6–1.1) | 0.9 (0.6–1.1) | 0.2 (0.1–0.4)* |
|  | 2.25 µM | 0.8 (0.6–1.0) | 0.9 (0.6–1.2) | 0.2 (0.1–0.4)* |
|  | 2.75 µM | 0.8 (0.7–1.1) | 1.0 (0.6–1.4) | 0.2 (0.1–0.3)* |
| MLN-4760 | 0.05 µM | 1.2 (1.0–1.4) | 1.2 (1.1–1.3) | 0.9 (0.7–1.1) |
|  | 0.1 µM | 1.0 (0.9–1.2) | 1.3 (1.1–1.5)* | 0.9 (0.8–1.2) |
|  | 0.25 µM | 0.9 (0.7–1.0) | 1.4 (1.3–1.6)* | 1.1 (0.8–1.2) |
|  | 0.5 µM | 1.1 (0.7–1.2) | 1.7 (1.6–1.7)* | 1.4 (1.4–1.7)* |
|  | 1 µM | 1.3 (1.1–1.6) | 1.7 (1.2–1.9)* | 2.0 (1.9–2.3)* |

Data are presented as median (min–max). *p-*values were calculated using the Mann–Whitney U test, comparing each concentration with the control group (cells incubated with medium without RAS inhibitors). * – *p*=0.029 vs control group, based on *n* = 4 independent experiments. FC – fold change = 2^−∆∆Ct^.

**Supplemental Table 5.** Effects of 20-HETE on cellular metabolic activity and GPR75 expression in HUVEC, RPTEC/TERT1, and HepG2 cells

| **20-HETE concen-tration** | **HUVEC metabolic activity (% of control), median (range)** | **HUVEC GPR75 expre-ssion (FC), median (range)** | **RPTEC/ TERT1 metabolic activity (% of control), median (range)** | **RPTEC/ TERT1 GPR75 expre-ssion (FC), median (range)** | **HepG2 metabolic activity (% of control), median (range)** | **HepG2 GPR75 expre-ssion (FC), median (range)** |
| --- | --- | --- | --- | --- | --- | --- |
| 1 nM | 94.3 (89.8–99.2)* | 1.3 (1.2–1.5)* | 101.5 (99.0–104.5) | 0.8 (0.6–1.1) | 84.2 (72.7–95.0)* | 1.2 (1.1–1.5)* |
| 2 nM | 94.1 (82.0–97.0)* | 1.4 (1.2–1.7)* | 97.9 (95.3–99.7)* | 0.9 (0.5–1.1) | 83.3 (73.9–84.5)* | 1.4 (1.1–1.5)* |
| 5 nM | 92.4 (80.5–97.2)* | 1.4 (1.2–1.6)* | 95.0 (90.3–98.6)* | 0.9 (0.7–1.1) | 81.8 (75.9–85.9)* | 1.5 (1.3–1.7)* |
| 10 nM | 88.5 (87.5–95.9)* | 1.5 (1.2–1.8)* | 91.8 (90.0–97.2)* | 1.2 (1.1–1.4)* | 76.3 (63.2–84.7)* | 1.5 (1.4–1.7)* |
| 100 nM | 80.7 (70.4–92.2)* | 1.6 (1.3–2.0)* | 79.0 (75.4–82.7)* | 1.3 (1.1–1.5)* | 74.8 (64.4–79.6)* | 1.7 (1.5–2.0)* |

Data are presented as median (min–max). *p-*values were calculated using the Mann–Whitney U test, comparing each concentration with the control group (cells incubated with medium without 20-HETE) * – *p*=0.029 vs control group, based on *n* = 4 independent experiments. FC – fold change = 2^−∆∆Ct^.

**Supplemental Figures**

**Supplemental Figure 1a**. Receiver operating characteristic (ROC) curve illustrating sensitivity and specificity of acute coronary syndromes (ACS) vs stable coronary artery disease (SCAD). Area under the ROC curve (AUC) = 0.91.

**Supplemental Figure 1b**. ROC curve illustrating sensitivity and specificity of ACS vs comparison. AUC = 0.925.

**Supplemental Figure 1c.** ROC curve illustrating sensitivity and specificity of ACS vs comparison + SCAD. AUC = 0.92.
